# Effect of Different Irrigants Applied After Post Space Preparation on Push-Out Bond Strength of a Self-Etch Resin Cement

**Published:** 2018-07

**Authors:** Hamid Jalali, Farzaneh Farid, Sudabeh Kulivand, Saeed Nokar, Kosar Dadgar

**Affiliations:** 1 Assistant Professor, Department of Prosthodontics, Tehran University of Medical Sciences, Tehran, Iran; 2 Postgraduate Student of Prosthodontics, Department of Prosthodontics, Tehran University of Medical Sciences, Tehran, Iran; 3 Associate Professor, Department of Prosthodontics, Tehran University of Medical Sciences, Tehran, Iran; 4 Assistant Professor, Department of Prosthodontics, Mazandaran University of Medical Sciences, Sari, Iran

**Keywords:** Dental Bonding, Tooth Root, Resin Cements, Post and Core Technique, Root Canal Irrigants, Sodium Hypochlorite, Chlorhexidine

## Abstract

**Objectives:**

The aim was to investigate the effect of intracanal irrigants and agents on the bond strength of intraradicular fiber posts to dentin.

**Materials and Methods:**

Root canals of 72 decoronated single-rooted premolars were cleaned, shaped, and obturated with gutta-percha and AH26. The specimens were incubated at 37°C and 100% humidity for seven days. Next, the coronal 10 mm of the canals were prepared to receive size 2 D.T. Light fiber post, and the specimens were randomly allocated into six groups according to the irrigants used after post space preparation: normal saline (control group), 5.25% sodium hypochlorite (NaOCl)/15 seconds, 17% ethylenediaminetetraacetic acid (EDTA)/60 seconds, 2% chlorhexidine (CHX)/5 minutes, MTAD/5 minutes, and acid-etching/15 seconds. All canals were rinsed with normal saline and dried. Fiber posts were cemented using Panavia F2.0. After 24 hours, two mid-root slices of 1mm thickness were obtained from each specimen. Push-out bond strength test was performed in a universal testing machine at 0.5mm/minute. The maximum applied load was recorded, and the bond strength was calculated in megapascal (MPa). Data were analyzed by one-way analysis of variance (ANOVA) and Tukey’s test.

**Results:**

The mean shear bond strengths of etched (53.21±12.11 MPa), MTAD (52.47±14.75 MPa) and EDTA (49.08±10.19 MPa) groups were significantly higher than that of the control group (39.82±10.04 MPa). The difference was marginally significant for CHX group (49.8±13.57 MPa) and not significant for NaOCl group (47.15±17.64 MPa).

**Conclusions:**

Etching or irrigating the root canals with MTAD or EDTA after post space preparation increases the bond strength of Panavia F2.0 to dentin.

## INTRODUCTION

In endodontically treated teeth with inadequate coronal structure, restorations are generally retained by intraradicular posts [1]. Whenever resin cements are used under repetitive occlusal loads, a strong and stable bond between the dentin, the luting cement, and the intraradicular post is a necessity for the longevity and success of this kind of restorations. The microstructure of dentin substrate and the type of adhesive system are two main factors that influence the bond strength between dentin and intraradicular posts [2,3]. Studies have shown a lower bond strength to radicular dentin than to coronal dentin for reasons such as a higher tubular density, high C-factor in the root region, and the influence of contraction stresses [3–5]. It has also been reported that agents and irrigants used during root canal therapy are able to affect the composition of radicular dentin. As a result, the capability of the adhesive system in making a strong bond is affected as well [5,6].

The process of cleaning and shaping of canals during root canal therapy causes the formation of a smear layer over the instrumented dentin [7]. Different irrigants and agents are used for complete removal of the smear layer that contains microorganisms and infectious deteriorated dentin. This step is an essential requirement for a successful prognosis of root canal therapy [7,8]. After the completion of endodontic treatment, if resin cements are used for cementing intraradicular posts, removal of the sealer-impregnated dentin is highly recommended in order to reach fresh dentin and make a hybrid layer [9–12]. As a result, once again, the smear layer forms on radicular dentin. However, this layer is rich in endodontic sealer and gutta-percha remnants plasticized by the generated heat [12,13]. This layer should either be removed or penetrated through by the adhesive system to reach intact dentin and make a strong bond [10–12]. Acid-etching removes the smear layer, while acidic primers or acidic self-adhesive systems dissolve the smear layer and incorporate it into the hybrid layer [14].

Farid et al [15] studied the effects of different sealers on the bond strength of resin cements. They found a significant reduction of retention in the posts cemented with Panavia F2.0 in canals obturated with AH26 sealer in comparison with the canals obturated using a zinc oxide eugenol (ZOE) sealer or the canals obturated without using a sealer [15]. Research shows that different factors might affect the ability of an adhesive system to penetrate the smear layer and reach intact dentin, including the type of the sealer [15,16], the thickness of the smear layer [11,12, 14], and the acidity of self-etch adhesive systems [3,14, 17]. According to the manufacturer, Panavia F2.0 is a self-etch resin cement with mild acidity of the adhesive system. Farid et al [15] stated that the mild acidity (pH≈2) [18] of the self-etch primer might not have been enough for penetrating the AH26-containing smear layer to reach intact dentin. Irrigating post spaces with chemical irrigants could remove the smear layer and increase the bond strength to radicular dentin. However, the results depend on the type of treatment, the duration of application, the radicular region, and the type of the adhesive system [19–23]. In the present study, the authors tested the effect of different canal irrigants used after post space preparation on the push-out bond strength of fiber posts cemented with Panavia F2.0 to radicular dentin. The null hypothesis was that different chemicals and agents would not affect the bond strength of the resin cement.

## MATERIALS AND METHODS

Seventy-two single-rooted premolars with the root length of 16–18 mm and with straight canals, without aberrant canal morphology or size, confirmed with radiography, were included in the present study. The exclusion criteria were large carious lesions, root cracks or resorption, more than 20-degree root curvatures at the apex, and former restorations or root canal therapy. The selected teeth were cleaned and disinfected in 0.5% chloramine-T solution and were stored in distilled water for later use within 3 months.

All the premolars were decoronated at 1mm coronal to the cementoenamel junction (CEJ) using diamond discs under heavy water irrigation. The canals were cleaned and shaped using nickel-titanium (Ni-Ti) rotary files size SX to F2 (ProTaper; Dentsply Maillefer, Ballaigues, Switzerland) and a low-speed handpiece (X-smart; Dentsply Maillefer, Ballaigues, Switzerland). The working length was determined by inserting a #10 k-file (Kerr, Sybron Endo, California, USA) inside the canal to a depth that its tip could be seen at the apical foramen. The canals were instrumented to 1 mm less than the actual canal length in order to prevent the extrusion of irrigants and agents. Between filings and after each file exchange, the canals were irrigated with 2 ml of 2.6% sodium hypochlorite (NaOCl; Chloraxid, Cerkamed, Poland). At the end of cleaning and shaping, all canals were irrigated with normal saline and dried. Obturation was performed by laterally condensing gutta-percha and sealer (AH26; Dentsply, DeTrey, Konstanz, Germany) in the canals. The canal orifice was covered with a eugenol-free temporary dressing (Cavit G; 3M ESPE, Seefeld, Germany). The specimens were kept in an incubator (Thermo Scientific^™^ Heratherm^™^, Thermo Fisher Scientific Inc., Waltham, MA, USA) at 37°C and 100% humidity.

After a week, the coronal 10 mm of the canals was prepared for the insertion of #2 fiber post (D.T. Light-Post system; Bisco, Inc., Schaumburg, IL, USA). Peeso reamers #2 (Dentsply Maillefer, Ballaigues, Switzerland) were used to remove gutta-percha from the canals. Black, red, and yellow drills provided by the manufacturer were used to shape the post spaces. Next, the specimens were randomly divided into 6 groups of 12 each, according to root canal treatment and irrigants used, as follows:

Group 1: Irrigation with 5 ml of normal saline
Group 2: Irrigation with 5 ml of 5.25% NaOCl (Chloraxid; Cerkamed, Poland) for 15 seconds
Group 3: Irrigation with 5 ml of 17% ethylenediaminetetraacetic acid (EDTA; Pulpdent; Watertown, MA, USA) for 60 seconds
Group 4: Irrigation with 5 ml of 2% chlorhexidine (CHX; Consepsis, Ultradent, South Jordan, UT, USA) for 5 minutes
Group 5: Irrigation with 5ml of MTAD (a mixture of tetracycline, citric acid, and detergent; Dentsply, Tulsa Dental, Tulsa, OK, USA) for 5 minutes
Group 6: Etching with 37% phosphoric acid (Total Etch; Ivoclar Vivadent AG, Schaan, Liechtenstein, Germany) for 15 seconds and rinsing with normal saline.

Size 25G Max-i-Probe needles (Dentsply, Tulsa Dental, Tulsa, OK, USA) were used for carrying the irrigants to the canals. The duration of the application of the agents was based on the manufacturer’s recommendation. After irrigation, all the canals were rinsed and dried by paper points, and the fiber posts were cemented with Panavia F2.0 (Kuraray Medical Inc., Tokyo, Japan) according to the manufacturer’s instructions. All the specimens were cemented at room temperature and were kept under finger pressure for 40 seconds and then at 37°C and 100% humidity for 24 hours.

**Fig. 1:**
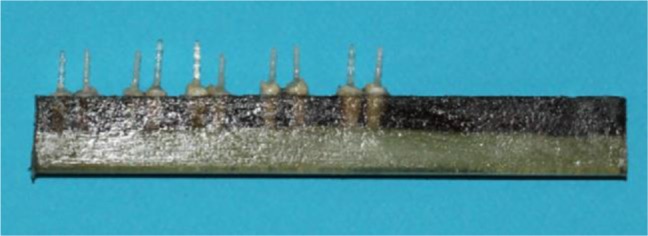
Mounting the samples in acrylic resin

For placing the specimens in a precision micro-cutting machine (Mecatome T 201 A; PERSI, France), the 4–5 mm apical part of each root was inserted vertically into a foam. The foam was placed inside a metal mold filled with acrylic resin (Ivoclar Vivadent AG, Schaan, Liechtenstein, Germany; Fig. 1).

**Fig. 2:**
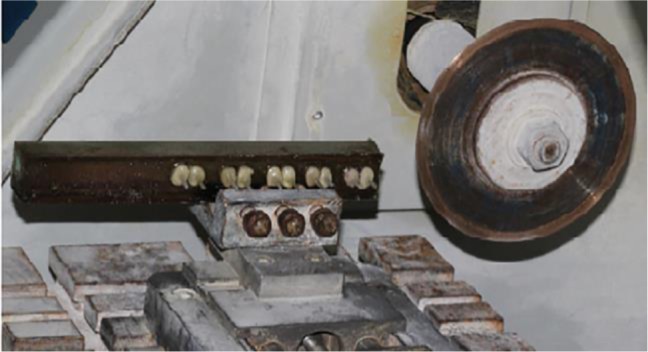
Sectioning of the mounted samples

After setting, the resin block with the embedded roots was separated from the mold and placed in the cutting machine (Fig. 2).

The machine cut the roots at a right angle to their long axes by a diamond disc under water cooling. Two slices of 1 mm thickness from the mid-root of each specimen were selected for the push-out test. The thickness of the slices was confirmed using a digital caliper with a 0.001-mm accuracy (Mitutoyo, Kanagawa, Japan). The coronal aspect of each slice was marked. Sections with uneven cement thickness or voids in the cement under 2× magnification were excluded.

**Fig. 3:**
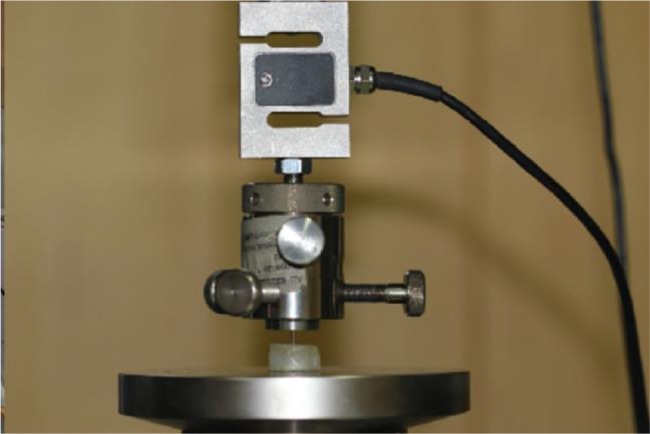
Push-out test

The push-out test was performed by placing the specimens on a special acrylic jig inside a Universal Testing Machine (Zwick-Roell, Ulm, Germany; Fig. 3).

A plunger of 1 mm diameter was used to load the post segment in each slice from the apical aspect, without contacting the surrounding dentin. The load was applied at 0.5mm/minute until the post was dislodged. The maximum load yielded by the post was recorded in Newton (N). The push-out value in Megapascal (MPa) was calculated using the following formula [20]:

$$\text{Push-out}\;\text{bond}\;\text{strength}\;=\;\text{F}/\text{A}=\;\frac{\text{maximum}\;\text{loaded}\;\text{force}}{\text{JI}(\text{R}1\;+\;\text{R}2)\;\sqrt{{\left(\text{R}1\;\text{-}\;\text{R}2\right)}^{2}}+\;{\text{h}}^{2}}$$

R1: radius of the post at the coronal aspect of the specimen
R2: radius of the post at the apical aspect of the specimen
h: the height of the slice

Data were analyzed by one-way analysis of variance (ANOVA) and Tukey’s honest significant difference (HSD) test using SPSS version 20 statistical software (IBM Co., Chicago, IL, USA).

## RESULTS

One-way ANOVA indicated a significant difference in the push-out bond strengths of EDTA, MTAD, and etched groups in comparison with the control group (P<0.05). The difference was marginally significant for CHX group (P=0.08). The bond strength of NaOCl group was higher than that of the control group, but the difference was not significant (P=0.73). The difference among the groups other than the control (groups 2 to 6) was not significant (P>0.9). The results are shown in Table 1.

**Table 1:** Mean bond strength (MPa) of Panavia F2.0 to dentin in experimental groups

**Group**	**Mean±SD**	**Minimum**	**Maximum**
Phosphoric acid	53.21±12.11	48.36	58.06
MTAD	52.47±14.75	46.84	58.64
EDTA	49.08±10.19	45.00	53.16
CHX	49.8±13.57	44.37	55.23
NaOCl	47.15±17.64	40.09	54.21
Normal saline	39.82±10.04	35.80	43.84

EDTA= Ethylenediaminetetraacetic Acid, CHX=Chlorhexidine, NaOCl=Sodium Hypochlorite, SD=Standard Deviation

## DISCUSSION

This study investigated the effect of post space treatment with common endodontic irrigants and etching on the bond strength of Panavia F2.0 to radicular dentin. The lowest bond was found in the control group, in which, the canals were irrigated with normal saline, whereas all other groups had higher bond strengths; therefore, the null hypothesis was rejected. Removal of the smear layer seems to be the most probable explanation for the positive effect of the chemical agents on the bond strength. However, according to Hayashi et al [19], removal of the smear layer is a disadvantage for the adhesion of the resin cement to radicular dentin when a self-etching system is used. They concluded that excessive demineralization caused by endodontic irrigation should be avoided when constructing a resin-dentin interface treated with self-etching adhesives [19]. They did not obturate the canals in their study, but in the present study, the canals were obturated with gutta-percha and an epoxy resin sealer (AH26). As a result, the composition of the smear layer in the two studies is different. This can explain the disagreement between the results of the two studies.

In the present study, the mean bond strength of NaOCl group was higher than that of the control group although the difference was not statistically significant. Regarding the effect of NaOCl, the results of studies are controversial.

Santos et al [22] reported the reduction of the bond strength of a self-etching adhesive to pulp chamber after irrigation with 10 ml of 5% NaOCl for 30 minutes, which was renewed every 3 minutes. The reduction of the bond strength can be attributed to the change of the redox potential of the bonding substrate due to oxygen release from residual NaOCl [19,20,22,24,25]. NaOCl is a nonspecific oxidizing agent [22,24, 25]. The long duration of application and a high concentration of NaOCl could result in incomplete polymerization of resin cements, which compromises the bond strength and decreases the elastic modulus and the flexural strength of human dentin [19,24]. Arisu et al [20] reported the lowest bond strength for the group in which post spaces were irrigated 60 seconds with 5 ml of 2.25% NaOCl. The bond strength was even lower compared to irrigation with distilled water [20]. On the other hand, Hayashi et al [19] reported an increased bond strength after irrigation with 10 ml of NaOCl for 15 seconds and explained it by the ability of NaOCl to remove the smear layer. In the present study, the canals were irrigated with 5.25% NaOCl for only 15 seconds. Since the duration of application was short, changes of the redox potential and reduction of the bond strength were not probable. At the same time, as the irrigant was not able to remove the smear layer, no significant increase in bond strength was observed. Therefore, in comparison with the control group, NaOCl neither reduced nor increased the bond strength. Irrigation with 5 ml of 17% EDTA for 60 seconds significantly increased the bond strength in the present study. The same is reported by Gu et al [26] and by Jacques and Hebling [27]. This positive effect could be attributed to the ability of EDTA to remove the smear layer. EDTA is a mild chelating agent [27] that selectively removes hydroxyapatite and non-collagenous proteins, avoiding major alterations of the native collagen fibrillar structure. However, in the study by Hayashi et al [19], in which, the canals were not obturated, irrigation with EDTA lowered the shear bond strength to dentin. The SEM observation in their study revealed collagen-rich and demineralized surfaces. They concluded that it may have been difficult to construct a firm adhesive interface between the resin cement and such demineralized radicular dentin [19]. Although the exposure of human root dentin to 17% EDTA for 2 hours completely eliminates calcium from the exposed surface to a depth of approximately 150 μm [23], short-term exposure, as in the present study, does not seem to have any negative effect on dentin.

In the present study, CHX had a marginally significant effect on the bond strength. CHX is a potent antiseptic that is widely used for plaque control in the oral cavity [8]. 2% CHX is used as a root canal irrigant [8]. CHX is unable to dissolve necrotic tissue remnants; therefore, it cannot be used as the main irrigant [8]. However, CHX is a matrix metalloproteinase inhibitor and prevents collagen degradation and disintegration at the bond interface over time [23]. Pelegrine et al [28] and Santos et al [22] reported a higher bond strength after irrigation with CHX in comparison with NaOCl or NaOCl plus EDTA. It is suggested that absorption of CHX by dentin may favor resin infiltration into dentinal tubules [22]. Pelegrine et al [28] and Vilanova et al [29] attributed the positive results of treating canals with 2% CHX gel to its viscosity, which might favor the mechanical cleansing of root canal walls thus promoting the effective removal of dentin debris and tissue remnants. These theories may explain the relatively high bond strength in the present study after irrigation with CHX.

In the present study, the bond strength in the canals irrigated with MTAD was significantly higher than that in the control group. MTAD is a mixture of doxycycline, acid citric, and Tween 80 (a detergent) [30]. It eliminates the inorganic smear layer and disinfects the root canal; however, some organic remnants of the smear layer still remain. Therefore, this agent is used as the final irrigant after NaOCl [30,31]. Yurdagüven et al [32] reported the reduction of the bond strength of Clearfil SE Bond to coronal dentin after MTAD application for 5 minutes. They concluded that a 5-minute application is too long. Their study was performed on coronal dentin; therefore, there was not any sealer-containing smear layer. This can explain the difference between the results of the above study and that of the present study.

Panavia F2.0 has a self-etch ED primer. Self-etch adhesives are more user-friendly than etch-and-rinse systems because they are less technique sensitive and have fewer application steps. Also, there is no risk of remaining of the etchant material inside root canals, no risk of over or under drying of the canal dentin, and no risk of discrepancy between the extent of demineralization and infiltration of adhesive [2,5,27,33]. After post space preparation, the self-etch primer has to dissolve the smear layer and incorporate it into the hybrid layer formed by demineralization of dentin and infiltration of monomers [27]. However, the thickness and buffering capability of the smear layer could hamper the primer to reach the intact dentin, compromising the bond strength [5,27, 33]. Although acid-etching significantly improved the bond strength in the present study, suggesting the addition of this step to the application process negates the purpose of developing self-etch adhesives.

Future studies are necessary to evaluate the long-term effects of these irrigants on the bond strength of different cements to dentin, especially under occlusal loads and thermal fluctuations.

## CONCLUSION

It can be concluded that irrigation of prepared post spaces with EDTA or MTAD or etching the canals with 37% phosphoric acid significantly increase the bond strength of Panavia F2.0 to dentin. The increase is marginally significant for CHX. However, irrigation with normal saline or NaOCl solutions has no positive effect on the bond strength.
